# P-2197. Pharmacist-Led Hepatitis C Virus Model of Care Effectively CURES Simplified Cases

**DOI:** 10.1093/ofid/ofae631.2351

**Published:** 2025-01-29

**Authors:** Lindsey Sheehan, Maribeth P Wright, Morgan Stacey, Anthony Fritz, Christian Rhudy, Daniel Moore

**Affiliations:** University of Kentucky HealthCare, Lexington, Kentucky; University of Kentucky Healthcare, Lexington, Kentucky; University of Kentucky HealthCare, Lexington, Kentucky; UK Healthcare/University of Kentucky, Wilmore, Kentucky; University of Kentucky HealthCare, Lexington, Kentucky; University of Kentucky, Lexington, Kentucky

## Abstract

**Background:**

Despite the existence of curative direct acting antiviral therapy, hepatitis C Virus (HCV) prevalence and mortality has increased in many regions of the United States. Existing models of care have failed to yield treatment rates needed for HCV elimination by 2030. In June 2023, we implemented UK-CURES (University of Kentucky - Cascade Utilizing Routinized screening through treatment to Eliminate Syndemics), a novel model of HCV care (Figure 1). This pharmacist-led model utilizes collaborative care agreements to carry out AASLD/IDSA best practices at the point-of-care in an effort to eliminate variation and waste along the HCV cascade.

**Figure 1.**
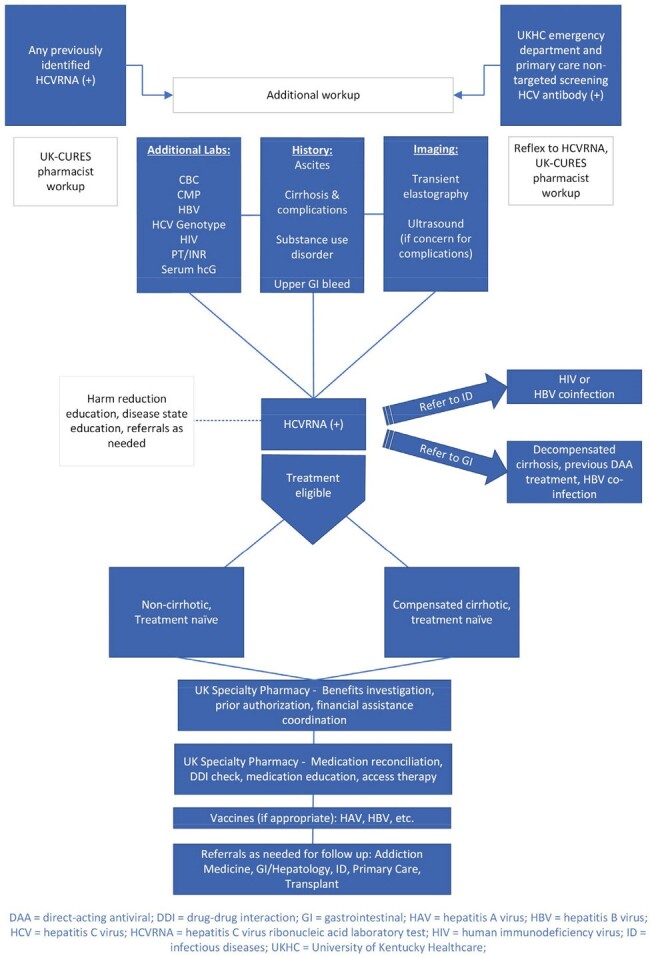
UK-CURES Simplified Treatment Algorithm

**Methods:**

This study is a retrospective descriptive analysis including patients evaluated by the UK-CURES team between 6/1/2023 and 5/1/2024. Patients were excluded from this analysis if they were not eligible for the UK-CURES HCV simplified treatment algorithm. Patient demographics, clinical and operational data were collected from electronic medical record modifications. Patients were considered lost to follow-up if they had not engaged with treatment 120 days and had not completed sustained virologic response (SVR12) laboratory results. Results are summarized with descriptive statistics.

**Figure:**
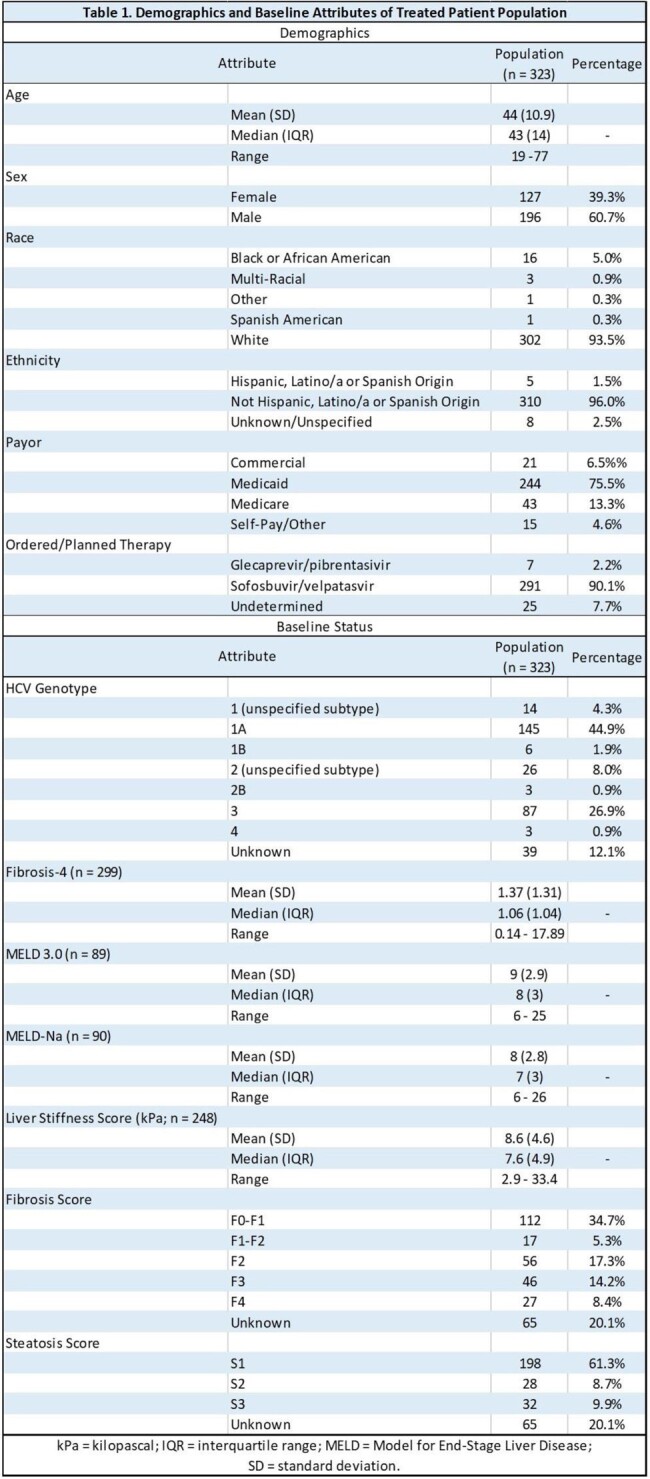

**Results:**

1,428 patients were evaluated by the UK-CURES team, of whom 1,105 were excluded from the treatment algorithm. The primary reason for exclusion was a negative confirmatory HCVRNA laboratory test (n = 579, 52.5%). Of 323 patients treated, demographics and baseline status are displayed in Table 1. Median time from first UK-CURES encounter to treatment order was 2 days, while the median time to first treatment dispense was 12 days (Table 2). Less than half of treated patients were lost to follow-up prior to obtaining SVR12 results (n = 147; 45.5%), and remaining patients are currently engaged in therapy (n = 141; 43.7%) or have completed SVR12 results (n = 35; 10.8%) (Figure 2). 32 patients (91%) were undetectable at SVR12.

**Figure 2.**
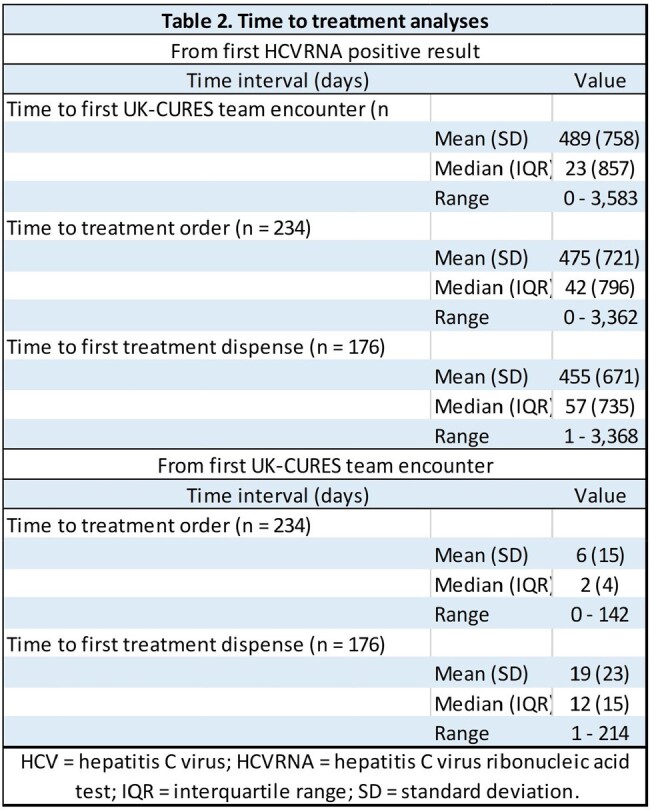
UK-CURES current patient status and attrition diagram

**Conclusion:**

Our novel pharmacist-led model of care, UK- CURES, improved key performance indicators along the HCV care cascade for simplified cases while maintaining best practices surrounding HCV guidelines. The success of this model has significant implications for future HCV elimination efforts.

**Figure:**
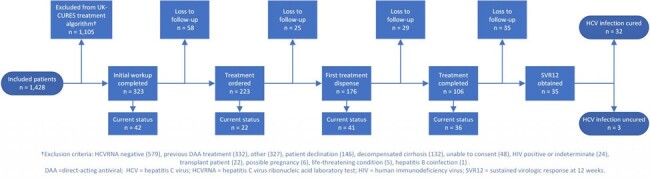

**Disclosures:**

Maribeth P. Wright, RPh, CSP, Gilead FOCUS Program: Gilead FOCUS grant support Christian Rhudy, PharmD, MBA, Amgen, Inc.: Grant/Research Support Daniel Moore, MD, MBA, FACEP, Gilead FOCUS: Grant/Research Support

